# Preoperative bacteriuria positivity on urinalysis increases wound complications in primary total hip arthroplasty regardless of the urine culture result

**DOI:** 10.1186/s12891-021-04725-4

**Published:** 2021-09-29

**Authors:** Linbo Peng, Yi Zeng, Yuangang Wu, Jing Yang, Fuxing Pei, Bin Shen

**Affiliations:** Department of Orthopedics, Orthopedic Research Institute, West China Hospital, Sichuan University, 37# Guoxue Road, Chengdu, 610041 Sichuan Province China

**Keywords:** Total hip Arthroplasty, Bacteriuria, Superficial wound infection, Prosthetic joint infection, Urinary tract infection

## Abstract

**Background:**

Current evidence does not recommend screening urine culture and curing asymptomatic bacteriuria (ASB) before joint arthroplasty. The bacteriuria count on pre-operative urinalysis is a more common clinical parameter. We aimed to investigate whether the bacteriuria count on preoperative urinalysis can increase postoperative wound complications in primary total hip arthroplasty (THA).

**Methods:**

We conducted a retrospective study that included patients who underwent primary THA in our institution from 2012 to 2018. We obtained preoperative urinalysis results before THA during the same hospitalization and identified patients with abnormal urinalysis. Receiver operating characteristic (ROC) curves were first generated to evaluate the predicted value of leukocyte esterase (LE), nitrite, bacteriuria, and pyuria in the urinalysis for superficial wound infection. Then, all included patients were divided into two groups according to the preoperative urinalysis: a bacteriuria-positive group and a bacteriuria-negative group. The primary outcome was the superficial wound infection rate within 3 months postoperatively, and the secondary outcomes included wound leakage, prosthetic joint infection (PJI), pulmonary infection, urinary tract infection (UTI), readmission rate within 3 months postoperatively, and length of stay (LOS) during hospitalization. We utilized univariable analyses to compare the outcomes between the two groups. A multivariable logistic regression model was generated to explore the potential association between bacteriuria and the risk of superficial wound infection, wound leakage, and readmission rate controlling for baseline values.

**Results:**

A total of 963 patients were included in the study. One hundred sixty patients had abnormal urinalysis. The AUCs for LE, nitrite, bacteriuria, and pyuria were 0.507 (95% confidence interval (CI), 0.315 to 0.698), 0.551 (0.347 to 0.756), 0.675 (0.467 to 0.882), and 0.529 (0.331 to 0.728), respectively. Bacteriuria was diagnostically superior to LE, nitrite, and pyuria. Among the 963 patients, 95 had a positive bacteriuria on preoperative urinalysis, and only 9 (9.5%) had a positive urine culture. Compared with the bacteriuria-negative group, the bacteriuria-positive group had a higher superficial wound infection rate (4.2% vs. 0.6%, *P* = 0.008), higher wound leakage rate (11.6% vs. 4.5%, *P* = 0.007), higher readmission rate (5.3% vs. 1.3%, *P* = 0.015) within 3 months postoperatively and longer LOS (6.19 ± 2.89 days vs. 5.58 ± 2.14 days, *P* = 0.011). After adjustment, the bacteriuria-positive group had a significantly increased risk of superficial wound infection (OR = 7.587, 95%CI: 2.002 to 28.755, *P* = 0.003), wound leakage (OR = 3.044, 95%CI: 1.461 to 6.342, P = 0.003), and readmission (OR = 4.410, 95%CI: 1.485 to 13.097, *P* = 0.008).

**Conclusion:**

Preoperative bacteriuria positivity on urinalysis significantly increased the risk of postoperative wound complications, readmission, and LOS in primary THA regardless of the result of the urine culture. Urinalysis is a fast and cost-acceptable test whose advantages have been underestimated.

**Level of evidence:**

Level III, observational study.

**Supplementary Information:**

The online version contains supplementary material available at 10.1186/s12891-021-04725-4.

## Introduction

Total hip arthroplasty is a successful surgical intervention that relieves pain and improves function for patients with end-stage arthritis of the hip joint [1, 2]. The number of THA surgeries has grown over the past few decades [3, 4]. It is estimated that the number of primary THA surgeries will reach 572 thousand by 2030 in the United States [5].

Wound complications are common after THA surgery [6, 7]. Postoperative wound complications, including superficial wound infection and wound leakage, may increase the risk of subsequent prosthetic joint infection (PJI) by up to 35-fold [8–10]. Although many risk factors for PJI have been identified, the risk factors for wound complications have not been established [11]. Recently, some studies have found that asymptomatic bacteriuria (ASB) is associated with an increased risk of PJI and wound complications [12–14].

Many surgeons regard screening and treating ASB before total hip arthroplasty as a standard protocol [15, 16]. British orthopaedic association guidelines recommend routine preoperative urine culture screening prior to joint arthroplasty [17]. Urine culture from a midstream, clean urine catch is the gold standard for diagnosing ASB [18]. However, urine culture is a time-consuming examination that requires at least 24–48 h to report the results [19]. In addition, the cost of urine culture is much higher than that of urinalysis [20, 21]. In recent years, some studies have found that most microorganisms obtained from surgery in PJI cases are unrelated to those found in preoperative urine culture, and preoperative antibiotic therapy cannot lower the PJI risk. Current evidence does not recommend screening urine culture and treating ASB before joint arthroplasty [13, 22].

Urinalysis is a more common test that can be used to screen many disorders [23]. Given that there are numerous unnecessary urine culture requests before THA surgery, we wondered whether abnormal urinalysis would increase the risk of postoperative complications of THA. Leukocyte esterase (LE), nitrite, bacteriuria count, and pyuria (WBC) are several indicators in urinalysis [24]. Among them, the most sensitive and specific parameter is bacteriuria count [20]. Given that the relationship between the bacteriuria count on preoperative urinalysis and postoperative complications have not been established, we performed a retrospective study to determine (1) the prevalence of abnormal urinalysis in primary THA patients; (2) the distribution of abnormal LE, nitrite, pyuria, and bacteriuria in abnormal urinalysis and their predictive value for superficial wound infection; and (3) whether the bacteriuria count on preoperative urinalysis is associated with a high risk of postoperative wound complications.

## Material and methods

### Study design

This retrospective study was approved by our institutional review board, and informed consent was obtained from each participant. We conducted a retrospective study including all consecutive patients who met the inclusion criteria from October 2012 to October 2018 in our institution. All patients underwent primary THA surgery by the same senior surgeon (B.S.). We obtained preoperative urinalysis results before THA surgery during the same hospitalization and identified patients with abnormal urinalysis.

After admission, all the patients were instructed to collect clean midstream urine samples correctly for the first time. Bacteriuria positivity was strictly defined as an isolation ≥2.3 *10^5^/ml (i.e., ≥ 230/μL) [25, 26]. Patients with bacteriuria positivity were taught to collect clean midstream urine samples again on the same day to avoid the possibility of contamination. Only patients who obtained two consecutive bacteriuria-positive results in the urinalysis were recorded as true bacteriuria-positive and underwent urine culture immediately. Otherwise, they were recorded as bacteriuria negative. We also recorded other parameters from the urinalysis, including LE, nitrite, and pyuria (WBC) [15, 27]. The LE and nitrite results were expressed as positive or negative [28, 29]. Pyuria was defined as positive with > 5/high-power lens (HP) [30, 31].

According to the Infectious Diseases Society of America, ASB was diagnosed as an isolation ≥10^5^ colony-forming units (CFUs)/mL in the absence of signs or symptoms of UTI on urine culture [27, 32]. All urine culture results were considered negative for isolations < 10^4^ CFUs/mL. When three or more different species of microorganisms were detected, we considered the results of urine culture to be contaminated [15]. The urine culture results were recorded as positive, negative, or contaminated. Once diagnosed with ASB by urine culture, patients were treated immediately with antibiotic therapy. We considered scheduling THA surgery only when these patients underwent another routine urinalysis and obtained a negative bacteriuria result.

### Inclusion and exclusion criteria

#### Inclusion criteria

1. Primary total hip arthroplasty;
2. Preoperative urinalysis results before THA surgery during the same hospitalization;
3. Absence of signs or symptoms of urinary tract infection (UTI), including urgency, frequency, dysuria, suprapubic, vaginal, urethral tenderness, and haematuria [33];
4. No infection or antibiotic use within 3 months before this hospitalization.

#### Exclusion criteria

1. Bilateral THA during the same hospitalization;
2. Femoral neck fracture;
3. Lack of adequate data.

### Outcome measures

We first analysed the distribution of different parameters in patients with an abnormal urinalysis. Receiver operating characteristic (ROC) curves were generated to evaluate the predictive value of LE, nitrite, bacteriuria, and pyuria in urinalysis for superficial wound infection. Because bacteriuria was the most sensitive and specific parameter in the urinalysis, patients were then divided into a bacteriuria-positive group and a bacteriuria-negative group. We collected variables including age, sex, body mass index (BMI), American Society of Anesthesiologists (ASA) score, diagnosis, comorbidity, preoperative haemoglobin (Hb), C-reactive protein (CRP), erythrocyte sedimentation rate (ESR), white blood cell (WBC) count and serum albumin. The primary outcome was the superficial wound infection rate within 3 months postoperatively. The clinical diagnosis of superficial wound infection was based on the Centers for Disease Control (CDC) criteria (supplement 1) [34]. Patients with superficial wound infection were treated with antibiotics or further surgical treatment. The secondary outcome was the wound leakage rate within 3 months postoperatively. Wound leakage was defined as leakage persisting for more than 3 days after surgery [35, 36]. Our study also included the PJI, pulmonary infection, UTI, readmission rates within 3 months postoperatively, and average length of hospital stay (LOS) during hospitalization.

### Statistical analysis

All data analyses and management were performed using SPSS 26.0 statistical software (IBM Corporation, USA). Categorical variables (sex, ASA score, diagnosis, comorbidity, superficial wound infection rate, wound leakage rate, PJI rate, pulmonary infection rate, UTI rate, any readmission rate) were presented as numbers (percentages), and continuous variables (age, BMI, Hb, CRP, ESR, WBC count, serum albumin, and LOS) were presented as the mean ± standard deviation. To determine the predictive value of LE, nitrite, bacteriuria, and pyuria, ROC curves were used to examine the relations between the true-positive rate (sensitivity) and false-positive rate (1-specificity) and to calculate the areas under the ROC curves (AUCs). Categorical variables were compared with the chi-square test (or Fisher’s exact test), and continuous variables were compared with Student’s independent-samples t-test. Then, the association between bacteriuria and the outcome (superficial wound infection, wound leakage, and readmission) was examined using multivariable logistic regression after adjusting for independent variables. For all the analyses, a *P*-value < 0.05 was considered statistically significant. All analyses were implemented with a two-tailed test.

## Results

### Study population and urinalysis outcomes

**Fig. 1:**
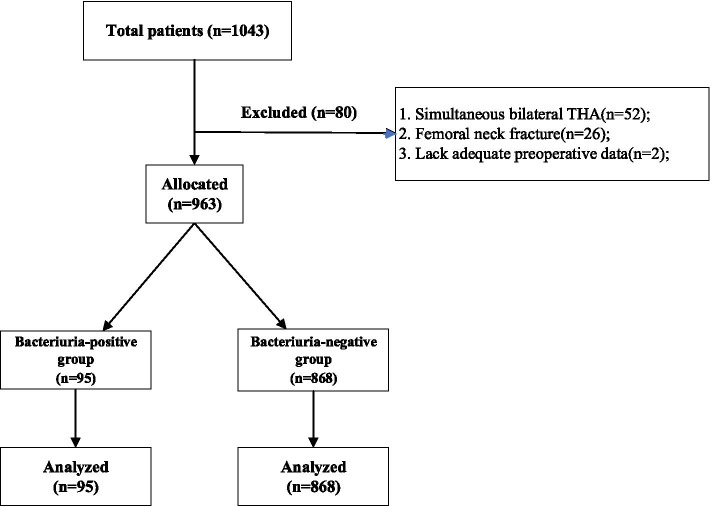
Flow diagram of patients involved in this study

From January 2012 to October 2018, 1043 patients were screened for eligibility. Eighty were excluded. Among them, 52 had simultaneous bilateral THA, 26 had femoral neck fractures, and 2 lacked adequate preoperative data (Fig. 1).

**Fig. 2:**
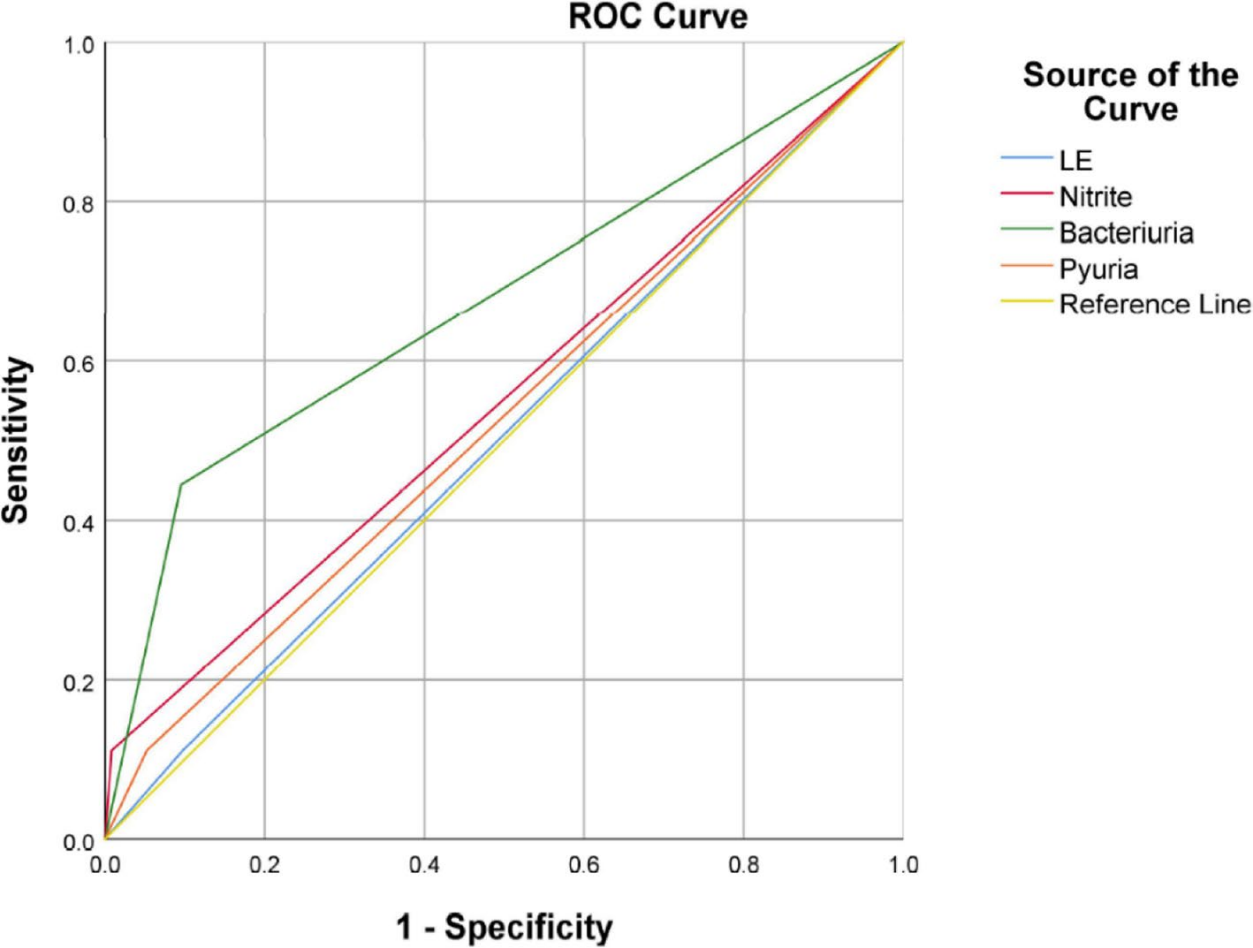
The ROC Curves of LE, Nitrite, Bacteriuria, and Pyuria. ROC, receiver operating characteristic; LE, leukocyte esterase

A total of 963 patients undergoing primary THA surgery were ultimately included in the study. One-hundred sixty had an abnormal urinalysis. The distributions of LE, nitrite, bacteriuria, and pyuria were shown in Table 1. Among the 160 patients with abnormal urinalysis, 94 were LE positive, 9 were nitrite positive, 95 were bacteriuria positive and 51 were pyuria positive. All the parameters in the urinalysis were represented in individual ROC curves to evaluate their predictive value for superficial wound infection. The AUCs for LE, nitrite, bacteriuria, and pyuria were 0.507 (95% confidence interval (CI), 0.315 to 0.698), 0.551 (0.347 to 0.756), 0.675 (0.467 to 0.882), and 0.529 (0.331 to 0.728), respectively. Bacteriuria was diagnostically superior to LE, nitrite, and pyuria (Fig. 2).

**Table 1:** the distribution of different parameters in abnormal urinalysis

Variable	Urinalysis positive	Urine culture positive	Superficial wound infection	Wound leakage	Pulmonary infection
LE (n)	94/963	8/94	1/94	5/94	1/94
Nitrite (n)	9/963	4/9	1/9	0/9	0/9
Bacteriuria (n)	95/963	9/95	4/95	11/95	1/95
Pyuria (n)	51/963	5/51	1/51	3/51	2/51

Leukocyte esterase, LE

There were 95 patients in the bacteriuria-positive group and 868 patients in the bacteriuria-negative group. The demographic characteristics of the two groups were shown in Table 2. The proportion of females among patients with bacteriuria positivity was 68.4%, which was significantly higher than patients with bacteriuria negativity (50.2%, *P* = 0.001). There were no significant differences between the two groups concerning age, BMI, ASA score, diagnosis, comorbidity, Hb, CRP, ESR, WBC count, nor serum albumin (Table 2).

**Table 2:** Demographic characteristics of the two groups

Variable	Bacteriuria-positive (*n* = 95)	Bacteriuria-negative (*n* = 868)	*P* Value
Age (year)^b^	54.22 ± 12.34	54.74 ± 12.40	0.697
Sex (Female)^a^	65(68.4%)	436 (50.2%)	0.001
BMI(kg/m^2^) ^b^	23.78 ± 3.49	23.37 ± 3.24	0.246
ASA score ≥ 3^a^	25 (30.5%)	203 (23.4%)	0.130
Diagnosis(n,%)			0.482
OA^a^	29 (30.5%)	195 (22.5%)	
DDH^a^	17(17.9%)	195 (22.5%)	
ONFH^a^	40(42.1%)	394 (45.4%)	
AS^a^	3(3.2%)	34 (3.9%)	
RA^a^	6(6.3%)	50 (5.8%)	
Comorbidity(n,%)			
Hypertension^a^	22 (23.2%)	202 (23.3%)	1.000
Diabetes^a^	7 (7.4%)	38 (4.4%)	0.197
Heart Disease^a^	3 (3.2%)	57 (6.6%)	0.263
COPD^a^	4 (4.2%)	13 (1.5%)	0.078
Laboratory test			
Hb(g/L)^b^	131.51 ± 15.63	133.45 ± 17.00	0.287
CRP (mg/L) ^b^	4.22 ± 3.01	4.45 ± 4.02	0.600
ESR (mm/h) ^b^	25.56 ± 20.18	28.20 ± 20.53	0.233
WBC count (× 10^9^) ^b^	5.93 ± 1.71	5.96 ± 1.79	0.892
Serum albumin (g/L) ^b^	43.48 ± 4.48	43.32 ± 3.79	0.701

BMI, Body mass index; American Society of Anesthesiologists, ASA; Osteoarthritis, OA; Developmental dysplasia of the hip, DDH; Osteonecrosis of the femoral head, ONFH; Ankylosing spondylitis, AS; Rheumatoid arthritis, RA; Chronic obstructive pulmonary disease, COPD; Hemoglobin, Hb; C-reactive protein, CRP; Erythrocyte Sedimentation Rate, ESR; White Blood Cell, WBC;

^a^ Compared with the Chi-square test (or Fisher exact test)

^b^ Compared with Student’s independent-samples t-test

### Urine culture outcomes

For all patients with bacteriuria positivity, urine culture was conducted immediately. Nine (9.5%) patients had a positive urine culture, 13 had a contaminated culture and 73 had a negative culture. The most common bacterial species in the urine culture was *Escherichia coli* (5 patients). We also collected some other bacterial species, including *Streptococcus milleri* (1 patient), *Candida glabrata* (1 patient), *Enterococcus faecalis* (1 patient), and *Klebsiella pneumoniae* (1 patient), in the urine culture. Of the 5 patients whose urine cultures were positive for *E. coli*, one of them had a superficial wound infection within 3 months postoperatively. Urine culture, bacterial species, and complications of the bacteriuria-positive group were shown in Table 3.

**Table 3:** Urine culture, bacterial species, and complication results of the bacteriuria positive group

Urinalysis	Urine culture	Bacterial species	Complication
Positive (n = 95)	Positive (9/95)	*Escherichia coli* (5)	WI(1); WL (1)
		*Streptococcus milleri* (1)	–
		*Candida glabrata* (1)	–
		*Enterococcus faecalis* (1)	–
		*Klebsiella pneumoniae* (1)	–
	Contaminated (13/95)	–	WI (1); WL (3)
	Negative (73/95)	–	WI (2); WL (7)

Superficial wound infection, WI; Wound leakage, WL

### Complications, readmission rate, and LOS

The mean superficial wound infection rate within 3 months postoperatively was significantly higher in the bacteriuria-positive group than in the bacteriuria-negative group (4.2% vs. 0.6%, *P* = 0.008). Similarly, the wound leakage rate was significantly higher in the bacteriuria-positive group than in the bacteriuria-negative group (11.6% vs. 4.5%, *P* = 0.007). There were no cases of PJI nor UTI 3 months postoperatively in the two groups. No difference was detected between the two groups in the pulmonary infection rate. The bacteriuria-positive group had a higher readmission rate than the bacteriuria-negative group (5.3% vs. 1.3%, *P* = 0.015). In addition, the LOS of bacteriuria-positive group was 6.19 ± 2.89 days, which was significantly longer than that of the bacteriuria-negative group (5.58 ± 2.14 days, *P* = 0.011). The results were shown in Table 4.

**Table 4:** Complication rate of the two groups in three months postoperatively

Variable	Bacteriuria-positive (n = 95)	Bacteriuria-negative (n = 868)	P Value
Superficial wound infection^a^	4(4.2%)	5(0.6%)	0.008
Wound leakage^a^	11(11.6%)	39(4.5%)	0.007
PJI^a^	0(0.0%)	0(0.0%)	N/A
Pulmonary infection^a^	1(1.1%)	10(1.2%)	1.000
UTI^a^	0(0.0%)	0(0.0%)	N/A
Any readmission^a^	5(5.3%)	11(1.3%)	0.015
Length of stay (days)^b^	6.19 ± 2.89	5.58 ± 2.14	0.011

Prosthetic Joint Infection, PJI; Urinary tract infection, UTI; N/A, Not Applicable

^a^ Compared with the Chi-square test (or Fisher exact test)

^b^ Compared with Student’s independent-samples t-test

After adjusting for independent variables with *P* < 0.5 and some other relative parameters in the regression model, including age, sex, BMI, ASA score, diagnosis, diabetes, heart disease, COPD, Hb, CRP, ESR, WBC, and serum albumin, the bacteriuria-positive group had a significantly increased risk of superficial wound infection (OR = 7.587, 95%CI: 2.002 to 28.755, *P* = 0.003, Table 5), wound leakage (OR = 3.044, 95%CI: 1.461 to 6.342, P = 0.003, Table 6), and readmission (OR = 4.410, 95%CI: 1.485 to 13.097, Table 7) relative to those who had negative bacteriuria results.

**Table 5:** Multivariable logistic regression on superficial wound infection

Factor	Superficial wound infection	
	OR (95% CI)	P Value
Bacteriuria-positive group	7.587 (2.002 to 28.755)	0.003
Bacteriuria-negative group	Reference	–

Adjusted for age, sex, BMI, ASA score, diagnosis, diabetes, heart disease, COPD, Hb, CRP, ESR, WBC, and serum albumin

**Table 6:** Multivariable logistic regression on wound leakage

Factor	Wound leakage	
	OR (95% CI)	P Value
Bacteriuria-positive group	3.044 (1.461 to 6.342)	0.003
Bacteriuria-negative group	Reference	–

Adjusted for age, sex, BMI, ASA score, diagnosis, diabetes, heart disease, COPD, Hb, CRP, ESR, WBC, and serum albumin

**Table 7:** Multivariable logistic regression on readmission

Factor	Any readmission	
	OR (95% CI)	P Value
Bacteriuria-positive group	4.410 (1.485 to 13.097)	0.008
Bacteriuria-negative group	Reference	–

Adjusted for age, sex, BMI, ASA score, diagnosis, diabetes, heart disease, COPD, Hb, CRP, ESR, WBC, and serum albumin

## Discussion

ASB has been confirmed to increase the risk of PJI and wound infection after joint arthroplasty [6, 12–14]. However, the diagnosis of ASB requires urine culture, which is a relatively expensive and time-wasting test [20]. Once diagnosed, ASB should be treated with relevant antibiotic that is often expensive, approximately £37 in the UK and €92 in Switzerland [16, 21, 37, 38]. Furthermore, some studies have found that most infectious microorganisms were unrelated to the previous urine culture results, and preoperative antibiotic therapy for ASB did not lower the postoperative PJI risk. They concluded that screening and treating ASB before joint arthroplasty was unnecessary [13, 14]. Given that there are numerous unnecessary urine culture requests before THA surgery, a reliable, cost-effective screening test is needed for the procedure.

This study conducted a retrospective analysis that included 963 patients who underwent primary THA from 2012 to 2018 and found a significant independent risk between preoperative bacteriuria positivity on urinalysis and postoperative wound complications, including superficial wound infection and wound leakage and readmission, within 3 months postoperatively. This was the first study, to our knowledge, to explore the relationship between preoperative bacteriuria positivity on urinalysis and postoperative complications for patients who had undergone primary total hip arthroplasty.

The prevalence of abnormal preoperative urinalysis is approximately 16.6% among all patients. Two studies reported an approximately 25% abnormal urinalysis rate in women, but they failed to explore this epidemiology in a wider age group [28, 39]. We considered the urinalysis abnormal if any of the following parameters were positive: LE, nitrite, pyuria, or bacteriuria. Four of 95 patients with abnormal bacteriuria had superficial wound infections in 3 months. Only one patient with abnormal LE, nitrite, or pyuria had superficial wound infections in 3 months. Eventually, bacteriuria was shown to be diagnostically superior to LE, nitrite, and pyuria for superficial wound infection according to the ROC curve analysis. Some previous studies found that negative LE and nitrite in the urinalysis excluded the presence of infection, but their high false-positive rates made them less effective [24, 40]. In our study, nitrite was closely associated with urine culture positivity. Four of 9 patients with positive nitrite were diagnosed with ASB by a positive urine culture. One of them had a wound infection within 3 months postoperatively.

Patients with two consecutive positive bacteriuria in the urinalysis were recorded as true bacteriuria-positive and underwent urine culture immediately. Only nine (9.5%) patients had positive urine culture results. Ollivere et al. found a 7% urine culture bacteriuria positivity rate, which was similar to ours [6]. The positive bacterial rate in urine culture varies greatly with age and sex [15]. In this study, the urine culture results of 13 (13.7%) patients were contaminated, while a previous study reported a contamination rate of 29–32% [41, 42]. We also found that *Escherichia coli* was the most common microorganism in the urine cultures, accounting for 55.6%. This is consistent with previous studies [43, 44].

We regarded superficial wound infection within 3 months postoperatively as the primary outcome. Superficial wound infection is difficult to diagnose [6]. Thus, we adopted the diagnostic criteria published by the CDC, which is consistent with previous studies [34, 45]. Continuous wound leakage increases the risk of postoperative PJI [7]. The bacteriuria-positive group had a significantly increased risk of wound leakage in our study. None of the patients experienced postoperative PJI within 3 months in the study. Given that the overall risk of PJI after primary total hip arthroplasty is approximately 0.4–1.5% [46, 47], there may be a false negative result for PJI in this study.

In our study, preoperative bacteriuria positivity on urinalysis was associated with higher superficial wound infection, wound leakage, and readmission rates, and a longer LOS. To the best of our knowledge, this was the first study to explore the relationship between preoperative bacteriuria positivity on urinalysis and postoperative complications in primary THA. Cordero-Ampuero and colleagues performed a prospective, randomized study of 471 patients who underwent THA and hemiarthroplasty surgery. Among the 13 patients who had an infection 3 months postoperatively, the microorganisms of the deep PJIs were different from those in the preoperative urine culture [15]. This suggests that ASB is not a direct source of PJI. However, it may undermine immune function and increase susceptibility to infection [16]. To reduce the interference of other risk factors for infection [48], we compared the demographic characteristics among the patients and found no significant difference in age, BMI, diagnosis, comorbidity, Hb, CRP, ESR, WBC, or serum albumin among the groups. The proportion of females in the bacteriuria-positive group was higher than that in the bacteriuria-negative group (68.4% vs. 50.2%, *P* = 0.000), which was similar to previous reports [6, 15, 37]. During wound healing, microorganisms may colonize wounds in small amounts and induce a superficial wound infection while the immune system is weakened [49]. We believe that preoperative bacteriuria positivity on urinalysis may be an independent predictive factor for poor wound healing and increase the rate of postoperative complications and readmission.

Our study has some limitations. First, this was a retrospective cohort study, and there may be some natural bias that cannot be avoided. Second, the sample size was relatively small, so we did not regard the PJI rate as the primary outcome in the study. However, the sample size was sufficient to explore wound complications, including superficial wound infections and wound leakage. Third, the standard cut-off for bacteriuria-positive count in urinary sediment analysis has not been established. Previous studies chose different cut-offs with different sensitivities, specificities, and standards of diagnosis for bacteriuria [50–54]. In our institution, bacteriuria positivity is determined for a count ≥2.3 *10^5^/ml (i.e., ≥ 230/μL). Similar to us, Broeren et al. affirmed that the cut-off for bacteriuria was 230/μL with a gold standard definition of < 10^5^ CFUs/ml and a sensitivity of 95% [25]. Finally, we found that bacteriuria positivity on urinalysis was a risk factor for postoperative wound complications in primary THA. We failed to investigate whether there were some interventions for bacteriuria that could reduce wound complications. All the patients with superficial wound infections were cured with antibiotics, and we did not assess wound secretion cultures. Therefore, we cannot determine whether the microorganisms responsible for the wound infection matched those in the preoperative urine culture.

## Conclusions

In conclusion, we found that preoperative bacteriuria positivity on urinalysis significantly increased postoperative wound complications, readmission rates, and length of hospital stay in primary THA regardless of the urine culture result. Urinalysis is a fast and cost-acceptable test whose advantages have been underestimated. To prevent wound complications, more attention should be given to patients with preoperative bacteriuria positivity before primary THA surgery.

## Supplementary Information

**Additional file 1.**

## Data Availability

The datasets used and/or analysed during the current study available from the corresponding author on reasonable request.
