# Supplementary material for: Preoperative bacteriuria positivity on urinalysis increases wound complications in primary total hip arthroplasty regardless of the urine culture result
Source: BMC Musculoskelet Disord. 2021 Sep 29;22:834. doi: 10.1186/s12891-021-04725-4 (PMC8480008; doi:10.1186/s12891-021-04725-4)
Supplement: Supplementary file 1 — Additional file 1. [file 12891_2021_4725_MOESM1_ESM.docx]

Supplement 1

The clinical diagnosis of superficial wound infection should be made by at least one of three criteria based on the CDC criteria.

| Criterion | Definition |
| --- | --- |
| 1 | Purulent drainage from the superficial infection |
| 2 | The superficial infection yields organisms from the culture of aseptically-aspirated fluid or tissue, or from a swab, and pus cells are present |
| 3 | At least two of the following symptoms and signs of inflammation:   1. pain or tenderness; b) localised swelling; c) redness; d) heat. |

CDC, Centers for Disease Control
